# Prompted Voiding Utilising Toilet Alarms and Individual Toileting Plans for Adults With an Intellectual Disability And Incontinence

**DOI:** 10.1111/jir.70149

**Published:** 2026-07-29

**Authors:** Janet Finlayson, Nick Gore, Dawn A. Skelton

**Affiliations:** ^1^ School of Health and Life Sciences Glasgow Caledonian University Glasgow UK; ^2^ Intellectual Disabilities Research Institute University of Birmingham Birmingham UK

**Keywords:** incontinence, intellectual disability, person‐centred planning, prompted voiding, toilet alarm, toileting support

## Abstract

**Background:**

Urinary incontinence is common amongst adults with an intellectual disability, yet there is a paucity of interventions to promote continence in this population. The aim was to test the feasibility of a toileting intervention with adults with an intellectual disability.

**Method:**

We conducted a feasibility study. Seventeen adults with an intellectual disability and incontinence participated in a 12‐week prompted voiding toileting support programme, which utilised wrist‐worn toilet alarms and individual plans to prompt regular toilet visits for voiding.

**Results:**

Thirteen (77%) followed their toileting plan over the 12‐week period with support, and 15 (88%) intended to continue with their plan beyond the study period.

**Conclusion:**

Prompted toilet visits, which incorporate individual toileting plans and meaningful personal goals‐setting, were found to be a feasible toileting intervention for the majority in the sample.

## 1 Introduction

Urinary incontinence (involuntary loss of urine) is common amongst adults with an intellectual disability, with prevalence rates estimated to be between 26% and 52% (van Timmeren et al. 2016; de Waal et al. 2009; Kroeger and Sorensen‐Burnworth 2009). Urinary incontinence is more common amongst adults with an intellectual disability than children with an intellectual disability (Kroeger and Sorensen‐Burnworth 2009). Whereas some adults, due to the severity or complexity of their intellectual disability, did not achieve urinary continence during childhood as a developmental milestone or thereafter (Kroeger and Sorensen‐Burnworth 2009), others have been continent previously but developed incontinence as adults (reference blinded for review, 2025) or have become incontinent because of frailty associated with older age (Keenan et al. 2024). This means that interventions that promote and/or maintain continence for adults with an intellectual disability, following assessment, are an important area for consideration.

Up until the 1970s, behavioural toilet training studies were conducted with adults with an intellectual disability living in long‐stay institutions, to promote continence through habit training (by encouraging regular and frequent toilet visits) (Kimbrell et al. 1967). The most well‐known of these studies was conducted by Azrin and Foxx (1971), using their rapid method of toilet training, whereby nine adults with a profound intellectual disability were given drinks every 30 min (to encourage the need to urinate), with scheduled toilet visits every 30 min (to prompt urinating in a toilet), daily for around 4 days. Incontinence was reduced immediately by 90% for these adults over the study period, but the method used punishment as well as positive reinforcements to achieve such a high rate of continence within a few days. For example, these adults were given positive edible and social reinforcements when they successfully voided in a toilet, but these reinforcements were withdrawn as a consequence when instances of incontinence occurred, and other forms of punishment were used to discourage further instances of incontinence, such as the participant being made to handwash their soiled clothing and/or mop or clean soiled areas on a chair or floor. Azrin and Foxx's (1971) study and similarly conducted studies are unethical by today's standards, as they involved aversive punishment strategies. Punishing and/or aversive strategies were not used in the current study which was grounded in person‐centred values and constructional practices, supportive of the rights and agency of people with an intellectual disability.

Since the 1970s, a number of toilet training interventions have been developed for mainly children with an intellectual disability and/or a diagnosed developmental disability (e.g., autism) (Vickers et al. 2021; Smith and Chaneb 2016; Kroeger and Sorensen‐Burnworth 2009). The interventions across these studies have mainly been modified versions of the rapid toilet training method, in that they incorporated scheduled toileting and prompted voiding, overhydration, behavioural reinforcement and overcorrection (Smith and Chaneb 2016; Kroeger and Sorensen‐Burnworth 2009). In addition, some of these interventions have included response restriction (e.g., preventing some behaviours to assert the target behaviour, e.g., not allowing the person to get up from the toilet seat until they have voided), using regular underwear without the use of incontinence pads, video modelling (showing the person videos to support toileting), gradual guidance and the use of toilet alarms (Smith and Chaneb 2016; Kroeger and Sorensen‐Burnworth 2009).

There has been a historical trend towards toilet training for children with an intellectual disability, rather than adults (Kroeger and Sorensen‐Burnworth 2009). This has led to a paucity of interventions and clinical guidelines to promote continence amongst adults with an intellectual disability. National Institute for Health and Care Excellence (2023) guidelines for treatment of urinary incontinence amongst adults with neurological conditions, which include but are not exclusive to adults with an intellectual disability. The current recommendation for the treatment of urinary incontinence, according to these guidelines, is behavioural toilet training, which considers prompted voiding and habit retraining. Prompted voiding and habit retraining refer to a technique of bladder and toilet habit training, whereby the person is prompted to urinate in a toilet at regular intervals, according to a predetermined schedule. A review of toilet alarms, which promote voiding and habit retraining, in toileting interventions for children with an intellectual disability found that, in all 12 studies reviewed, instances of incontinence decreased and instances of successful voiding increased for all or most participants (Levato et al. 2016). Since the 1970s, the use of toilet alarms as prompts/reminders in conjunction with behavioural toilet training has been championed for use with children with an intellectual disability, but no previous studies have been conducted with adults with an intellectual disability. The aim of this research, therefore, was to explore the feasibility of a prompted voiding and habit retraining intervention for adults with an intellectual disability with urinary incontinence, with the aid of toilet alarms, and individual toileting plans to support participation.

## 2 Method

### 2.1 Participants and Procedure

Adults with an intellectual disability and incontinence were recruited into this study via community intellectual disability health care team caseloads across National Health Service (NHS) Greater Glasgow and Clyde and NHS Lanarkshire. The inclusion criteria were person that has urinary incontinence and person that is able to sit on a toilet (with or without support) for up to 3 min. People were not excluded if they also had bowel incontinence or behaviours that challenge. All participants were advised that they would require assistance from a supportive person (a relative or support worker), to support their participation in this study. Twenty‐two adults who met this inclusion criteria completed an incontinence and toileting issues assessment with their supporter, during a home visit by a researcher; 20 of whom, at the same time, also completed urine sample analysis to screen for possible urinary tract infections (UTIs). The results of the incontinence and toileting issues assessments, and urine analyses, have been published separately (Finlayson, Skelton, et al. 2025; Finlayson, Gore, et al. 2025).

Two of the 22 adults with an intellectual disability were withdrawn from the study prior to this intervention phase: one woman with an intellectual disability due to the family supporter she lives with being too unwell to support her participation and one autistic man with an intellectual disability because his toileting issues—selecting to urinate and defecate on a floor rather than in a toilet—were not considered to have been amenable to this prompted voiding in a toilet intervention. A further three participants withdrew within the first few weeks of commencing their individual toileting plan: one adult due to issues around their supported living team not being able to support participation; one adult due to family illness; and one autistic man with an intellectual disability, who showed no interest in following his toileting plan, and whose instances of incontinence, his support staff later stated, were mainly in response to changes in his routine, such as being supported by a new support worker (and were unlikely to be amenable to a prompted voiding in a toilet intervention).

Seventeen adults with an intellectual disability completed this 12‐week toileting intervention. Their characteristics are reported in Table 1. Two of these participants had an existing behavioural support plan (not related to toileting), and their psychologists were contacted beforehand (with each person's or their nearest relative's consent) to ensure participation would not interfere with their existing plan.

**TABLE 1:** Sample characteristics.

	Sample *n* = 17 (100%)
Age	Mean 42 years
	Standard deviation 16.9
	Range 18–65 years
Sex	Male 6 (35%)
	Female 11 (65%)
Lives with …	Lives alone, independently 1 (6%)
	Lives with family 10 (58%)
	Supported living—individual tenancy 3 (18%)
	Supported living—group tenancy 3 (18%)
ID cause	Down syndrome 2 (12%)
	Unknown 13 (76%)
	Birth injury 2 (12%)
Level of ID	Mild 2 (12%)
	Moderate 8 (47%)
	Severe 7 (41%)
Mobility	Walks independently 9 (53%)
	Walking stick or frame 4 (23%)
	Wheelchair user (outdoors only) 3 (18%)
	Wheelchair user (indoors & outdoors) 1 (6%)
Do you have autism?	Yes 6 (35%)
	No 11 (65%)
Do you have epilepsy?	Yes 3 (18%)
	No 14 (82%)
Do you have cerebral palsy?	Yes 3 (18%)
	No 14 (82%)
Do you have a visual impairment?	Yes 6 (35%)
	No 11 (65%)
Do you have a hearing impairment?	Yes 3 (18%)
	No 14 (82%)
Do you have bowel incontinence?	Yes 6 (35%)
	No 14 (82%)
Do you experience incontinence during the night?	Yes 13 (76%)
	No (24%)

### 2.2 Project Materials

For the intervention, participants and their supporters were asked to follow an individualised toileting plan, including the participant wearing a toilet alarm to prompt scheduled toilet visits over a 12‐week assessment period. The latter was the standard intervention across all participants. Each participant, with support from their supporter, provided information during their home visit assessment to inform the development of their individual toileting plan. This was to ensure their toileting plan was personalised according to their individual needs, wishes and preferences in line with person‐centred practices.

#### 2.2.1 Individual Toileting Plan

The individual toileting plan for each participant was developed as an accessible document, according to the person's preferred communication style and other preferences, including pictures, photographs or Makaton symbols, preferred words and easy language. The pictures, photographs or symbols were selected with the person and their supporter to ensure they were appropriate and familiar to them (this included ensuring characters featured in pictures/symbols represented them, according to their sex, ethnicity, physical characteristics, etc.). Each toileting plan was developed by the researcher in partnership with the adult with an intellectual disability and their supporter and was reviewed by a psychologist on the research team to ensure it was clinically appropriate to implement.

The toileting protocol was divided into different sections and individualised for each participant accordingly. The front page contained the person's name, toileting plan start and end dates, and a privacy statement (to confirm that the plan was private and only for people who support the person going to the toilet to see). The second section listed the individual's personal toileting goals (e.g., to have less toileting accidents), and the third section described the person's toilet alarm, which included instructions for what to do when the alarm sounded. The fourth section described the person's preferred responses (e.g., verbal support/reassurance) to them (i) voiding in a toilet successfully, (ii) sitting on a toilet but not voiding, (iii) instances of ‘toilet accidents’ (incontinence) and (iv) the person choosing not to take part in their toileting plan for whatever reason, at any time on any day. Fifth section described what the person needed to help them with (i) using a toilet, (ii) sitting on a toilet and (iii) accessing and using a toilet when they are away from home. This was to capture any supports from another person or aids or adaptations they needed to support their toileting plan. Sixth section added ‘my toileting tips’ the person intended to follow to promote continence (e.g., trying to drink less caffeinated drinks), and Seventh section contained ‘important advice for me’ so the person and their supporter knew who could help them to continue with and review their toileting plan after the study period (e.g., their community health care professional). A copy of the individual toileting plan protocol is available in the Supporting Information.

Participants and their supporters were given an accessible toileting guide, developed by the research team, which provided useful information and guidance about the following: how the bowel and bladder work; diet and fluid advice (e.g., recommended daily fluid intake); sitting on the toilet (e.g., optimal sitting position); getting to and using the toilet; and the toilet environment. Participants and their supporters were encouraged to consider this information and guidance when completing Sixth section of their toileting plan.

#### 2.2.2 Toilet Alarms

Each participant was given the choice of using one of the following toilet alarms, based on their toileting needs and preferences:

1. Toilet alarm prompts inputted into their own personal mobile phones.
2. A vibrating Malem Vibro Watch, which is a wrist‐worn watch with programmable alarm prompts to schedule toilet visits.
3. A Malem wearable toilet alarm, which is worn discretely in a person's underwear, interrupts instances of incontinence to encourage the person to void the remaining amount in a toilet. This toilet alarm may also be used with a Malem bed mat during the night, which is also available to any participant choosing this option.

Information about these Malem products is available via their website (https://malemmedical.com/).

#### 2.2.3 Outcome Measures

Participants and their supporters completed questionnaires at 6‐ and 12‐week intervals, to learn about their self/proxy reported issues and outcomes, of following their prompted voiding in a toilet plans over the 12‐week assessment period. Participants and their supporters also completed 72‐h toilet charts at baseline (prior to commencing their toileting plan), 6 weeks, and at the end of the 12‐week period, to record and be able to compare (1) number of successful voids in a toilet and (2) number of instances of incontinence over these 72‐h periods.

### 2.3 Analysis

Data were entered into an IBM SPSS Version 31 dataset to produce frequency and descriptive statistics. Qualitative data collected via open‐ended questions was categorised and reported according to frequency of responses.

## 3 Results

### 3.1 Sample

The majority of adults with an intellectual disability who took part in this intervention had a mild to moderate intellectual disability (10 people, 59%), and six adults (35%) had bowel incontinence as well as urine incontinence. Six of the adults with an intellectual disability (25%) were also autistic (Table 1).

### 3.2 Individual Toileting Plans

All 17 adults used a Malem Vibro Watch during the 12‐week assessment period, based on their individual needs and preferences, with an average number of 10 toilet visit scheduled prompt/reminders per day (Table 2). All participants followed their individual toileting plan during the day only (Table 2), despite 13 (76%) also experiencing incontinence during the night (Table 1). These participants did not want their sleep disturbed by toileting prompts during the night.

**TABLE 2:** Summary of content of 17 individual toileting plans.

	*N* = 17
My preferred word for urine is …	Wee 8
	Toilet 3
	Pee 2
	Pee‐pee 2
	Wee‐wee 1
	Number one 1
My preferred word for faeces is …	Poo 8
	Toilet 3
	Dirty/messy 2
	Number two 2
	Poo‐poo 1
	Jobby 1
My preferred word for incontinence is …	Accident 10
	Being wet/wetting 5
	Poo‐poo in it 1
	‘Can I get cleaned?’ 1
My preferred word for continence is …	Being/staying dry 11
	No specific word for continence 4
	Being/staying clean 2
My toileting plan is for …	Daytime use only 17
My toilet training goals are …	Fewer toileting accidents 13
	Less reliance on pads 13
	More regular & successful toilet visits 6
	Increased independence in toileting 6
	Better communicate my need to use the toilet 4
	Reduce my anxiety around toileting 2
	Less disruption to my daily routine 2
	Overcome my distraction 1
	Initiate unprompted toilet visits more 1
	Reduce the need to change my soiled clothes 1
	Stop hiding my soiled clothes 1
	Fewer urinary tract infections 1
	Less need for catheter use 1
When I sit on and use the toilet successfully, please say/do …	Nothing specific 1
	First part of sentence …
	Well done … 11
	Brilliant … 2
	Good job/man … 2
	Amazing … 1
	… Second part of sentence
	… That's brilliant/fantastic/great 7
	… You're doing really well 2
	… Well done 2
	… You are a star 1
	… Good man 1
	… Good job 1
	… You get a nice pat on the back 1
	… Your family are/will be proud of you 1
When I sit on the toilet but do not manage to use it, please say/do …	Nothing specific 2
	First part of sentence …
	Not to worry … 6
	Never mind … 2
	That's okay … 2
	Do not stress … 1
	No rush … 1
	There you go … 1
	We've started … 1
	Let us see … 1
	… Second part of sentence
	… Let us try again later 7
	… Everything is okay 2
	… Well done for trying 1
	… Take your time 1
	… You're doing really well 1
	… You tried really hard 1
	… Just breathe 1
	… Let us wait and see if we can finish 1
When I have a toilet accident, please say/do …	First part of sentence …
	Do not worry … 9
	It's okay … 5
	Do not stress … 1
	These things happen … 1
	Smile (nonverbal response) 1
	… Second part of sentence
	… Let us get you clean and changed 6
	… It's not your fault 5
	… These things happen 3
	… Nobody is cross with you 2
	… That's life, let us get you sorted 1
	… I'm here to help 1
At times when I choose not to take part in my toilet training plan, please say/do …	First part of sentence …
	It's okay … 6
	It's your choice … 6
	Not to worry … 1
	… Second part of sentence
	… Let us try again later 10
	… It's there to help you 1
	… It's your choice 1
	… Let us see how you feel later 1
	… You're doing really well 1
	General reminder (no specific phrase)
	Remind me the purpose of my watch is to help me 1
	Remind me gently the plan is for my independence 1
	Remind me that the plan is there to help me 1
Things I need to help me are …	Being kind/reassurance to help me feel less anxious 4
	Walking aid, e.g., walking frame 4
	Help to clean myself 2
	Letting me know you are here to help 2
	Using a room spray so the bathroom smells nice 1
	Remind me to respond to my watch alarm 1
	15‐min countdown to visit the toilet 1
	Flatulence means I need to poo 1
	Remind me to pull my clothes down fully 1
	Reminder when I am too preoccupied to visit a toilet 1
	Stairlift to reach the bathroom 1
	Hoist to transfer to a toilet 1
	Shower chair over a toilet 1
	Having my favourite music or toys in the bathroom 1
	Toilet visits at the same times every day 1
	Being able to hold, touch or fiddle with objects 1
	Holding my watch for me if I do not want to wear it 1
My toilet training tips are …	Try to drink 6 to 8 drinks per day 12
	Drink more water 10
	Drink less caffeinated drinks 3
	Drink less fizzy (carbonated) drinks 2
	Drink decaffeinated tea and coffee 1
Number of toilet alarm prompts per day	Mean 10
	Range 5–12
	Standard deviation 2.1

The participants' preferred words, toileting goals, preferred responses, toileting support needs and tips, which were incorporated into each of their individual toileting plans, are described in Table 2. For most participants (13 people, 76%), their supported toileting plan goals were to have fewer toileting accidents (instances of incontinence) and to become less reliant on pads for incontinence (13 people, 76%).

#### 3.2.1 Issues and Outcomes

Any issues that the participants and their supporters experienced, in following their individual toileting plan and schedule, were self/proxy reported at 6 and 12 weeks (Table 3). Three participants and their supporters reported worsening in their nighttime incontinence at 6 weeks (which they said was due to increasing their fluid intake) but not 12 weeks. One other participant was experiencing a worsening in their daytime incontinence at 6 weeks but not 12 weeks for the same reason. The main issues reported for some participants at both 6‐week (six people, 35%) and 12‐week (12 people, 70%) follow‐up were related to wearing their toileting alarm watch (e.g., being reluctant or forgetting to wear it or watch malfunction). In terms of self/proxy reported outcomes at 6 and 12 weeks, these were wide‐ranging (Table 3), from experiencing fewer toilet accidents (five people, 29%) at 6 weeks, to finding the watch prompts helpful (nine people, 53%) and experiencing less night time incontinence (three people, 18%) at 12 weeks.

**TABLE 3:** Self‐proxy reported issues and outcomes at 6‐ and 12‐week follow‐ups.

	6‐week follow‐up	12‐week follow‐up
Have you been following your plan over the past 6 weeks?	Yes 10 (59%)	Yes 13 (77%)
	No 6 (35%)	No 4 (23%)
	Missing 1 (6%)	Missing 0 (0%)
How happy or unhappy are you with your plan?	Very happy 5 (29%)	Very happy 6 (35%)
	Happy 6 (35%)	Happy 10 (59%)
	Neutral 3 (18%)	Neutral 1 (6%)
	Very unhappy 1 (6%)	Very unhappy 0 (0%)
	Missing 2 (12%)	Missing 0 (0%)
How happy or unhappy are you with your toilet alarm?	Very happy 5 (29%)	Very happy 5 (29%)
	Happy 4 (23%)	Happy 6 (35%)
	Neutral 2 (12%)	Neutral 3 (18%)
	Unhappy 3 (18%)	Unhappy 2 (12%)
	Very unhappy 1 (6%)	Very unhappy 1 (6%)
	Missing 2 (12%)	Missing 0 (0%)
How happy are you that you are working towards your toileting goals?	Very happy 4 (23%)	Very happy 6 (36%)
	Happy 6 (35%)	Happy 10 (58%)
	Neutral 4 (35%)	Neutral 1 (6%)
	Unhappy 1 (6%)	Unhappy 0 (0%)
	Missing 2 (12%)	Missing 0 (0%)
Self‐reported issues at 6‐week follow‐up	Incontinence has become worse during the night 3 (18%)	
	Reluctant to wear watch 1 (6%)	
	Self‐conscious about wearing watch 1 (6%)	
	Watch malfunction 1 (6%)	
	Forgets to wear watch 1 (6%)	
	Person was initially nervous about wearing watch 1 (6%)	
	Does not like feel of the watch on their skin 1 (6%)	
	Person views plan as interfering with their daily routine 1 (6%)	
	Person finds reminders and prompts challenging 1 (6%)	
	Incontinence worse because of diet and fluid intake 1 (6%)	
	Person was in hospital during assessment period 1 (6%)	
Self‐reported outcomes at 6‐week follow‐up	Fewer toilet accidents 5 (29%)	
	Less reliance on pads 2 (12%)	
	Has been able to go on a family holiday again 1 (6%)	
	Has become more independent 1 (6%)	
	Less anxious about leaving the house 1 (6%)	
	Active participation in plan and toilet monitoring 1 (6%)	
	No incontinence meant not having to hide soiled clothes 1 (6%)	
Self‐reported issues at 12‐week follow‐up	Watch malfunction 6 (35%)	
	Refuses to wear watch or does not like wearing 5 (29%)	
	Watch not suitable for person (not enough stimulus) 1 (6%)	
	Still incontinent when feeling anxious 1 (6%)	
	Incontinence dependent on mood (e.g., tiredness) 1 (6%)	
	No change in bowel incontinence 1 (6%)	
Self‐reported outcomes at 12‐week follow‐up	Watch prompts are helpful 9 (53%)	
	Less night time incontinence 3 (18%)	
	Less incontinence 2 (11%)	
	Support staff more aware of toileting needs 2 (11%)	
	Enjoyed wearing watch 2 (11%)	
	More independence 1 (6%)	
	Less reliance on pads 1 (6%)	
	Less bowel incontinence 1 (6%)	
	Less incontinence due to drinking less fluids 1 (6%)	
	Person enjoyed setting goals and following plan 1 (6%)	
	Person liked watch (technology in general) 1 (6%)	
	More toilet visits 1 (6%)	
	More assistance provided in the toilet 1 (6%)	
	Less urgency 1 (6%)	

Thirteen (77%) of the participants and their supporters reported that they had been following their toileting plan in the last 6 weeks of the assessment period (which was an increase from 10 people, 59% in the first 6 weeks), and 16 (94%) said that they were very happy/happy with their toileting plan at the end of 12 weeks (which was an increase from 11 people, 64% at the end of the first 6 weeks). In addition, 16 (94%) said they were very happy/happy that they were working towards their toileting goals (which was an increase from 10 people, 58% at the end of the first 6 weeks) (Table 3). At the end of the study period, 15 (88%) participants and their supporters said that they intended to continue with their individual toileting plan.

### 3.3 Objective Versus Self/Proxy‐Reported Outcomes

The toileting charts demonstrated the following total number of instances of successfully voiding in a toilet over a 72‐h period: mean 11 instances at baseline (ranging between 0 and 30 instances, standard deviation [SD] 9.2); mean 12 instances at 6‐week follow‐up (ranging between 0 and 28 instances, SD 9.3); and mean 13 instances at 12‐week follow‐up (ranging between 0 and 30 instances, SD 9.8). Table 4 compares the level of agreement between self/proxy reported successful voiding in a toilet outcomes at 6 and 12 weeks, with 72‐h toilet chart data. Although only one participant (6%) and their supporter reported that they were experiencing fewer instances of successful voiding in a toilet at the end of the 12‐week period, the level of agreement between self/proxy reported and toilet chart data for the same period was only reached for nine participants (53%).

**TABLE 4:** Level of agreement about instances of successful voiding in a toilet between self/proxy reported outcomes and 72‐h toilet charts.

Have you/the person you support had more instances of successful voiding in a toilet?	Self‐reported at 6‐week follow‐up	72‐h toilet chart at 6‐week follow‐up^a^	Self‐reported at 12‐week follow‐up	72‐h toilet chart at 12‐week follow‐up^b^
Yes, more	12 (70%)	5 (29%)	8 (48%)	6 (35%)
About the same	3 (18%)	5 (29%)	8 (48%)	2 (12%)
No, less	0 (0%)	4 (24%)	1 (6%)	6 (35%)
Missing	2 (12%)	3 (18%)	0 (0%)	3 (18%)
Level of agreement	Agreement 7 (41%)		Agreement 9 (53%)	
	Disagreement 7 (41%)		Disagreement 5 (29%)	
	Missing 3 (18%)		Missing 3 (18%)	

^a^ Compared against baseline 72‐h toilet chart data.

^b^ Compared against 6‐week 72‐h toilet chart data.

The toileting charts demonstrated the following total number of instances of incontinence recorded over a 72‐h period: mean 6 instances at baseline (ranging between 0 and 15 instances, standard deviation (SD) 5.5); mean 7 instances at 6‐week follow‐up (ranging between 0 and 19 instances, SD 6.9); and mean 5 instances at 12‐week follow‐up (ranging between 0 and 19, SD 6.3). Table 5 compares the level of agreement between self/proxy reported outcomes at 6 and 12 weeks, with 72‐h toilet chart data. Although only one participant (6%) and their supporter reported that they were experiencing more instances of incontinence at the end of the 12‐week period, the level of agreement between self/proxy reported and toilet chart data for the same period was only reached for nine participants (53%).

**TABLE 5:** Level of agreement about instances of incontinence between self/proxy reported outcomes and 72‐h toilet charts.

Have you/the person you support had fewer instances of incontinence?	Self‐reported at 6‐week follow‐up	72‐h toilet chart at 6‐week follow‐up^a^	Self‐reported at 12‐week follow‐up	72‐h toilet chart at 12‐week follow‐up^b^
No, more	6 (35%)	6 (35%)	1 (6%)	4 (24%)
About the same	3 (18%)	5 (29%)	4 (23%)	4 (24%)
Yes, less	5 (29%)	3 (18%)	12 (71%)	9 (52%)
Missing	3 (18%)	3 (18%)	0 (0%)	0 (0%)
Level of agreement	Agreement 5 (29%)	Agreement 9 (53%)	Agreement 5 (29%)	Agreement 9 (53%)
	Disagreement 9 (53%)	Disagreement 8 (47%)	Disagreement 9 (53%)	Disagreement 8 (47%)
	Missing 3 (18%)	Missing 0 (0%)	Missing 3 (18%)	Missing 0 (0%)

^a^ Compared against baseline 72‐h toilet chart data.

^b^ Compared against 6‐week 72‐h toilet chart data.

## 4 Discussion

### 4.1 Adherence and Usability

The aim of this study was to explore the feasibility of prompted voiding, utilising toilet alarms and individual toilet plans, as an intervention for adults with an intellectual disability and urinary incontinence. This was found to be feasible for the majority of participants with a mild to severe intellectual disability, with 13 (77%) following their toilet plan over the 12‐week assessment period with support and 15 (88%) intending to continue with their individual plan beyond the study period. Out of the initial preintervention sample of 22 adults with an intellectual disability, 8 (36%) of participants with an intellectual disability were also autistic; 6 of whom were recruited into this intervention; and 2 of whom were withdrawn and for whom the intervention was not considered appropriate to address their particular toileting issues (selectively urinating and defecating on a floor rather than in a toilet or being incontinent in response to a change in their routine). In these instances, functional assessment and multicomponent behaviour support planning around the person's toileting issues and needs would likely have been more appropriate (National Institute for Health and Care Excellence 2018; National Institute for Health and Care Excellence 2025).

In terms of issues, 11 (64%) participants and their supporters reported that they were very happy/happy with wearing their toilet alarm at 12‐week follow‐up. However, six (36%) reported they were neutral/unhappy/very unhappy wearing their toilet alarm at 12 weeks. Reasons given for this at 6‐ and 12‐week follow‐up included being reluctant, too self‐conscious or nervous to wear their watch; forgetting or refusing to wear; watch malfunction; or the watch not providing enough stimulus for the person to use as a toilet reminder. The toilet alarm was used as an aid to promote scheduled toilet visits during this intervention, and other aids or the use of no aids at all may also be considered for future interventions.

### 4.2 Comparison With Previous Literature

There are no recent studies with community‐based adults with an intellectual disability available to compare our findings. Unlike Azin and Foxxes' (1971) rapid toilet training method study for example, which included aversive/punitive strategies, this intervention promotes progressive, rather than immediate, improvements towards fewer instances of incontinence through a person‐centred and constructional approach supportive of the rights and agency of adults with an intellectual disability (and does not use any punitive/aversive strategies). The personalised features of the plans and personal goal setting to support this prompted voiding intervention were important for individuals with an intellectual disability to meaningfully engage with their toileting plan and for their supporters to support them consistently according to their individual needs, preferences and goals. Inclusion of these features has not been reported elsewhere in the toileting literature. The rapid toilet training method (1971) involved drinking fluids at 30‐min intervals, as well scheduled toilet visits every 30 min. We did not include the former as a core component of this intervention, although we did give participants and their supporters advice on their recommended daily fluid intake; this may explain why three (18%) of participants experienced an increase in instances of incontinence, through drinking more fluids than usual, in the first 6 weeks of the assessment period.

### 4.3 Outcomes

Self/proxy reported outcomes at both 6‐ and 12‐week follow‐up were wide ranging, but mainly: fewer toilet accidents (five, 29%) and less reliance on pads (two, 12%) at 6 weeks; and watch prompts being helpful (nine, 53%) and less night time incontinence (three, 18%) at 12 weeks (Table 3). These outcomes can be compared with the participants' personal goals (Table 2), to determine whether participants were progressing towards their personal goals through following their individual toilet plans. Seventeen adults and their supporters reported that they were very happy/happy that they were working towards their goals at 12‐week follow‐up. Other self/proxy reported outcomes, not previously stated as personal goals, were being able to go on holiday with family again (one, 6%) and being less anxious about leaving the house (one, 6%) at 6‐week follow‐up; and support staff being more aware of toileting needs (two, 11%) and experiencing less bowel incontinence (one, 6%) at 12‐week follow‐up. These self/proxy reported outcomes are indicative of improvements in the person's quality of life and wellbeing. Future research could develop this further, by utilising quality of life assessment (e.g., Patrick et al. 1999) alongside self/proxy reported outcomes. Self‐reported data collected from the person's supportive carer would also be useful for determining the impact of participation in the intervention on the person's overall support (e.g., carer burden).

Eight (48%) proxy/self‐reported that they were experiencing more instances of successful voiding in a toilet by the end of the 12‐week period, but this was only reported for 6 adults (35%) via their 72‐h toileting charts. The level of agreement between both measures at 12‐week follow‐up was 53% (for nine adults). Twelve adults (71%) proxy/self‐reported that they were experiencing fewer instances of incontinences by the end of the 12‐week period, but this was only reported for nine adults (52%) via their 72‐h toileting charts. The level of agreement between both measures at 12‐week follow‐up was 53% (for nine adults). The discrepancies between proxy/self‐reported data and objective toileting chart data may be due in part to missing data, as it was not always clear whether incomplete charts signified data that were missing or data recorded as a zero (for no instances of successful voiding or incontinence). The feasibility of gathering objective data needs careful consideration in future research. Future research should, for example, ensure there are clear instructions for participants and their supporters completing toileting charts, to be able to distinguish between missing data and zero‐recorded entries, and, in order to objectively measure effectiveness, collect toileting chart data over a longer assessment period (7 days instead of 3 days), and provide daily telephone reminders for participants to complete the charts. A single‐subject research design may also be useful for future research studies, to determine meaningful outcomes within an individual support planning framework.

### 4.4 Strengths and Limitations

The main strength is that this is the first published study of an intervention to reduce instances of incontinence and promote continence, amongst community‐based adults with an intellectual disability with urinary incontinence, for decades. It is also noteworthy that this study was grounded in person‐centred and constructional practices that have not been the focus of prior toileting literature. The main limitations are the small exploratory sample size of 17 adults with an intellectual disability and discrepancies between proxy/self‐reported data and toileting chart data. Larger samples for future studies will be required, with further consideration of strategies to support data collection. We were only able to recruit 22 adults with an intellectual disability for the whole study over a 9‐month recruitment period, which was 73% of our target sample size of 30. A longer participant recruitment period over multiple sites will be required for larger studies to achieve the target sample size. Only 2 out of 19 participants withdrew during this intervention, so the retention of 17 (89%) of participants was a strength.

## 5 Conclusion

Urinary incontinence is a common but underresearched concern for adults with an intellectual disability. Individualised toilet plans, which support prompted toilet visits, are a feasible intervention for adults with an intellectual disability with incontinence, with support from their relatives or support workers. A research and practice focus on incontinence amongst adults with an intellectual disability is required to further develop our understanding of the most effective strategies and interventions to promote and evaluate fewer instances of incontinence and progress towards continence for adults with an intellectual disability who experience incontinence.

## Funding

This work was supported by the Baily Thomas Charitable Fund.

## Ethics Statement

This study was ethically approved by the National Scotland A Research Ethics Committee. In keeping with the Adults with Incapacity (Scotland) Act 2000 (Scottish Government 2000), five adults with an intellectual disability gave their own consent to participate, and the nearest relative of 12 adults with an intellectual disability gave their consent with the person on their behalf, and the person was supported to participate as much as they were able to.

## Conflicts of Interest

The authors declare no conflicts of interest.

## Supporting information




**Data S1:** Supporting Information

## Data Availability

Anonymised data are available directly from the research team.
