# Supplementary material for: Prompted Voiding Utilising Toilet Alarms and Individual Toileting Plans for Adults With an Intellectual Disability And Incontinence
Source: J Intellect Disabil Res. 2026 Jul 29;70(11):1179–91. doi: 10.1111/jir.70149 (PMC13630586; doi:10.1111/jir.70149)
Supplement: Supplementary file 1 [file JIR-70-1179-s001.docx]

**INDIVIDUAL TOILETING PLAN**

**PROTOCOL**

**Please Note: This protocol includes 3 individual toileting plan examples.**

**COMPLETION AND IMPLEMENTATION**

The individual toileting plan must be completed by the researcher, following discussion about content with the person with a learning disability, and their supporter.

Researcher’s satisfactory assessment of the person’s incontinence and toileting needs must be reviewed and agreed by the research team before their individual toileting plan is implemented. This includes review of data on the person’s daily fluid intake and toileting pattern over 72 hours at baseline.

The person’s individual toileting plan must be reviewed and agreed by the psychologist on the research team before it is implemented.

The person’s GP (and psychologist/psychiatrist/behaviour specialist, where applicable) must be consulted, with the person’s consent, before their individual toileting plan can be implemented.

**ACCESSIBILITY:**

The individual toileting plan must be in an accessible format for the person with a learning disability It is recommended that accessible information features easy language, the use of pictures, photographs, and/or symbols. The toileting plan must:

- Use words and pictures/photographs/symbols which are familiar and meaningful to the person.
- Only use words and images which are positive.
- Use images which are relevant to the person (e.g. ethnicity, man or woman, wheelchair user).
- Use font and image sizes which are suitable for the person’s vision status.

The images featured may be used to provide laminated prompt cards or signage, to assist the person’s toileting plan if they want them. These should be offered to the person as an option.

**CONTENT:**

The individual toileting plan must contain the following seven sections:

***1. Front Page:***

The front page which will include the person’s name, information about the start and end dates of their plan, and a privacy statement.

***2. My Goals:***

This section will list the person’s toileting goals, described in terms of more instances of voiding in a toilet successfully, and fewer instances of ‘toilet accidents’ or incontinence. Becoming continent should not be listed as a goal at any time, to avoid negative feelings associated with not being able to achieve goals within a 12-week period.

***3. My Toilet Alarm:***

The person’s type of toilet alarm must be described in this section, and include instructions on what to support the person to do when the alarm sounds. There should be two sections for any person who will be using a different type of toilet alarm during the night; one section named ‘My Daytime Toilet Alarm’, and one section named ‘My Night Time Toilet Alarm’.

***4. My Personal Responses:***

This section will document the words and/or images the person would use to describe 1) incontinence, and 2) continence.

This section will state the person’s preferred responses to each of the following:

- Voiding in the toilet successfully.
- Sitting on the toilet but not voiding.
- Instances of ‘toilet accidents’.
- The person choosing not to take part in their plan, for whatever reason, at any time on any day.

Each personally preferred response must be positive (e.g. motivating and encouraging).

***5. Things I Need to Help Me:***

This section will document any help or aids the person will need to help them 1) use a toilet, 2) sit on a toilet seat for about three minutes, and 3) use a toilet when they are out.

***6. My Toileting Tips:***

This section can be used to document any tips the person can try and follow, to help promote continence. The ‘Toilet and Toileting Guide’ can be used as a guide for completing this section.

***7. Important Advice for Me:***

This section will contain mandatory support advice for all participants, about choosing to continue following their personal toilet training plans beyond the 12-week assessment period.

**PERSONAL TOILETING PLAN**

**Anne Anonymous (Example 1)**

**This is my personal toilet training plan**

**This is my toilet training plan for 12 weeks**

**Start Date: Monday, 13th July 2020**

**End Date: Sunday, 4th October 2020**

**This plan is private. It is only for people who support me with going to the toilet to see**

**MY GOALS**

**This plan is to help me try and use a toilet more often**

**This plan is to help me try and pee in a toilet more often**

**This plan is to help me try and poo in a toilet more often**

**This plan is to help me try and not need to use pads as much**

**MY TOILET ALARM**

**It is important to know I am wearing a toilet alarm in my pants**

**When my toilet alarm sounds…**

**I have started doing the toilet**

**And I need help immediately to sit on a toilet to finish what I am doing there**

**I do not wear my toilet alarm at night**

**I do not want to be woken up to use the toilet when I am sleeping**

**MY PERSONAL RESPONSES**

**The words I use to describe continence are staying dry or staying clean**

**The words I use to describe incontinence are wetting my pad or soiling my pad**

**These are my preferred positive responses…**

**When I sit on and use a toilet successfully. Please say, ‘Well done’**

**When I sit on a toilet but do not manage to use it. Please say, ‘Well done for sitting on the toilet’**

**When I have a toilet accident. Please say, ‘These things happen’**

**When I wet or soil my pad. Please say, ‘Never mind. Next time’**

**At times when I choose not to take part in my toileting plan. Please say, ‘That’s okay. Next time’**

**THINGS I NEED TO HELP ME…**

**To use a toilet: A raised toilet seat and grab rails**

**Sit on a toilet for about 3 minutes: Reading my favourite book or magazine about cats**

**To use a toilet when I am out: Knowing where the toilets are. Having my radar key with me. Having my ‘can’t wait’ card with me to avoid toilet queues**

**MY TOILETING TIPS**

**I can try and have 6 to 8 drinks every day**

**IMPORTANT ADVICE FOR ME**

**My toilet training plan is for 12 weeks. If I choose to keep following my toilet training plan for more than 12 weeks, I know it is important to tell a health professional (for example, a nurse or psychologist) in my local community learning disability team about this.**

**I know the health professional will be able to give me advice or support, to help me to stop using my toilet alarm over time.**

**I know the researcher can tell me how to contact my local community learning disability team.**

**PERSONAL TOILETING PLAN**

**John Smith (Example 2)**

**This is my personal toileting plan**

**This is my toilet training plan for 12 weeks**

**Start Date: Monday, 13th July 2020**

**End Date: Sunday, 4th October 2020**

**This plan is private. It is only for people who support me to see**

**MY GOALS**

**This plan is to help me try and visit a toilet more often**

**This plan is to help me try and urinate in a toilet more often**

**This plan is to help me try and not have toilet accidents**

**MY TOILET ALARM**

**It is important to know I am wearing a toilet alarm watch on my wrist**

**When my toilet alarm watch vibrates…**

**It is reminding me to go to the toilet**

**I know to go to the toilet when my toilet alarm watch vibrates**

**I know to go to the toilet any time I need to urinate (not just when my toilet alarm watch vibrates)**

**I do not wear my toilet alarm watch when I am sleeping**

**MY PERSONAL RESPONSES**

**The words I use to describe continence are continence**

**The words I use to describe incontinence are wetting myself**

**These are my preferred positive responses…**

**When I visit a toilet on my own I need privacy.**

**When I tell you I have visited a toilet but not urinated successfully. Please say nothing and nod.**

**When I tell you I have visited a toilet and urinated successfully. Please say nothing and nod and smile.**

**When I have a toilet accident. Please say nothing but ask me if I need help to clean myself and change my clothes.**

**At times when I choose not to take part in my toilet training plan. Please say, ‘No problem. It’s up to you when you want to wear your watch or not’.**

**THINGS I NEED TO HELP ME…**

**To use a toilet: Privacy and patience. Please don’t knock the toilet door when I am using it**

**It is important to always have my favourite Imperial Leather soap in the toilet for me to wash my hands**

**Try and use a toilet for about 3 minutes: Counting 3 minutes with my watch**

**To use a toilet when I am out: Having hand sanitizer with me. I do not like washing my hands in public toilets which might be dirty**

**MY TOILET TRAINING TIPS**

**I can try and drink less cans of coca cola**

**I can try and drink more water and glasses of fresh orange juice**

**IMPORTANT ADVICE FOR ME**

**My toilet training plan is for 12 weeks. If I choose to keep following my toilet training plan for more than 12 weeks, I know it is important to tell a health professional (for example, a nurse or psychologist) in my local community learning disability team about this.**

**I know the health professional will be able to give me advice or support, to help me to stop using my toilet alarm over time.**

**I know the researcher can tell me how to contact my local community learning disability team.**

**PERSONAL TOILET TRAINING SUPPORT PLAN**

**Jenny Wren (Example 3)**

**This is my personal toileting plan**

**This is my toilet training plan for 12 weeks**

**Start Date: Monday, 13th July 2020**

**End Date: Sunday, 4th October 2020**

**This plan is private. It is only for people who support me to use the toilet see**

**MY GOALS**

**This plan is to help me try and do a number 1 in a toilet more often**

**This plan is to help me try and do a number 2 in a toilet more often**

**This plan is to help me try and have a dry pad more often**

**MY DAYTIME TOILET ALARM**

**It is important to know I am wearing a toilet alarm in my pants**

**When my toilet alarm watch sounds…**

**I have started doing the toilet**

**And I need to go to the toilet immediately to finish what I am doing there**

**I also need to go to the toilet any time I point to my bathroom picture card**

**MY NIGHT TIME TOILET ALARM**

**It is important to know my toilet alarm plugs into my bed mat sensor when I am sleeping**

**My bed mat sensor is on my bed**

**It is important to know I put my toilet alarm on my bedside table when I am sleeping**

**I do not wear it in my pants when I am in bed**

**When my toilet alarm sounds…**

**I have started doing the toilet**

**And I need to go to the toilet immediately to finish what I am doing there**

**I am happy to wake up during the night to use the toilet if I need to**

**MY PERSONAL RESPONSES**

**The words I use to describe continence are being clean**

**The words I use to describe incontinence are doing a number 1 in my pants or doing a number 2 in my pants**

**These are my preferred positive responses…**

**When I use my bathroom picture card to ask to go to the toilet. Please give me a high-five and say, ‘Good job, you asked to go to the toilet’.**

**When I sit on use a toilet successfully. Please give me a high-five and say, ‘good job, you used the toilet’.**

**When I sit on a toilet but do not use it successfully. Please pat my shoulder and say, ‘Good job, you sat on the toilet’.**

**When I have a toilet accident. Please pat my shoulder and say, ‘It’s okay’.**

**When my pad or bed is wet or soiled. Please pat my shoulder and say, ‘It’s okay’.**

**At times when I choose not to take part in my toilet training plan. Please say, ‘Good job, you deserve a day off. It’s okay’.**

**THINGS I NEED TO HELP ME…**

**To use a toilet: Assistance from another person to transfer from my wheelchair to the toilet, and hand rails**

**It is important for me to use a toilet step to help me sit in a good position on the toilet**

**It is important to remember to move my toilet step out of the way. Before I am ready to stand up and move back into my wheelchair**

**Try and use a toilet for about 3 minutes: Sing or listen to my favourite songs**

**To use a toilet when I am out: Having my radar key with me to use a toilet for wheelchair users**

**Having my headphones with me so I can listen to music when I am sitting on a toilet**

**MY TOILET TRAINING TIPS**

**I can practise having a good sitting position on the toilet. I can practise putting my elbows on my knees**

**IMPORTANT ADVICE FOR ME**

**My toilet training plan is for 12 weeks. If I choose to keep following my toilet training plan for more than 12 weeks, I know it is important to tell a health professional (for example, a nurse or psychologist) in my local community learning disability team about this.**

**I know the health professional will be able to give me advice or support, to help me to stop using my toilet alarm over time.**

**I know the researcher can tell me how to contact my local community learning disability team.**
